# Phthalates and Non-Phthalate Plasticizers and Thyroid Dysfunction: Current Evidence and Novel Strategies to Reduce Their Spread in Food Industry and Environment

**DOI:** 10.3390/toxics13030222

**Published:** 2025-03-19

**Authors:** Francesca Gorini, Alessandro Tonacci, Chiara Sanmartin, Francesca Venturi

**Affiliations:** 1 Institute of Clinical Physiology, National Research Council, 56124 Pisa, Italy; alessandro.tonacci@cnr.it; 2 Department of Agriculture, Food and Environment, University of Pisa, 56124 Pisa, Italy; chiara.sanmartin@unipi.it (C.S.); francesca.venturi@unipi.it (F.V.)

**Keywords:** thyroid, plasticizers, phthalates, alternative plasticizers, food contact materials, food packaging, chemical migration, chemical leaching, endocrine disruption, environmental exposure

## Abstract

Thyroid hormones (THs) play a crucial role in various biological functions, including metabolism, cell growth, and nervous system development, and any alteration involving the structure of the thyroid gland and TH secretion may result in thyroid disease. Growing evidence suggests that phthalate plasticizers, which are commonly used in a wide range of products (e.g., food packaging materials, children’s toys, cosmetics, medical devices), can impact thyroid function, primarily affecting serum levels of THs and TH-related gene expression. Like phthalate compounds, recently introduced alternative plasticizers can leach from their source material into the environment, particularly into foods, although so far only a very limited number of studies have investigated their thyroid toxicity. This review aimed at summarizing the current knowledge on the role of phthalate and non-phthalate plasticizers in thyroid dysfunction and disease, describing the major biological mechanisms underlying this relationship. We will also focus on the food industry as one of the main players for the massive spread of such compounds in the human body, in turn conveyed by edible compounds. Given the increasing worldwide use of plasticizers and the essential role of THs in humans, novel strategies should be envisaged to reduce this burden on the thyroid and, in general, on human health.

## 1. Introduction

Thyroid hormones (THs) have long been established to play a fundamental role in normal fetal growth and development of almost all tissues, primarily the central nervous system, and in regulating energy homeostasis in adults [1,2]. Therefore, any alteration of thyroid function, even if modest, may lead to neurocognitive impairment and interfere with lipid and glucose metabolism, blood pressure, and body weight, ultimately resulting in the onset or exacerbation of components of the metabolic syndrome [3,4]. Thyroid disease is a comprehensive term including a variety of pathological conditions affecting the physiology and/or morphology of the thyroid gland including hypothyroidism, hyperthyroidism, autoimmune thyroid diseases, thyroid nodules, and thyroid cancer (TC) [5]. It has been estimated that around 200 million people globally are diagnosed each year with thyroid disease, and of these, 0.2–1.3% of individuals living in iodine-sufficient areas present overt hyperthyroidism, while the prevalence of overt hypothyroidism in the general population varies between 0.2% and 5.3% in Europe and between 0.3% and 3.7% in the United States [5,6]. On the other hand, although most countries have introduced iodized salt programs that have substantially reduced the prevalence of iodine deficiency disorders over the past 30 years, approximately 2 billion people (equivalent to 35–40% of the world population) are currently at risk of iodine deficiency, a trace element essential for the synthesis of TH thyroxine (T4) and the active hormone 3,5,3′-triiodothyronine (T3), 50 million of whom have clinical manifestations [7,8,9,10]. Autoimmune thyroid diseases, which encompass a broad range of phenotypes collectively characterized by antibodies directed against thyroid antigens, affect 2–5% of the general population, with a higher prevalence among women than men [11]. The prevalence of thyroid nodules may reach 66% in the general population, with rates varying depending on the detection methods (ultrasound and/or autopsy) and with female sex, older age, and body mass index considered as the main risk factors [12]. Although thyroid nodules represent a high disease burden, only 10–15% are malignant, namely TC, which can give rise to metastasis and have a poor prognosis for patients [13]. Despite the supposed overdiagnosis due to advancements in diagnostics, the incidence of TC has increased in all continents except Africa, probably due to a lower possibility of access to ultrasound and imaging examinations [10]. TC is currently ranked as the 13th most diagnosed cancer in the United States, and, in 2020, the worldwide age-standardized incidence rates of TC were 10.1 per 10,000 women and 3.1 per 10,000 men [14,15].

In addition to sex, age, race/ethnicity, genetic susceptibility, smoking, and iodine intake, exposure to endocrine disrupting chemicals (EDCs), natural or synthetic compounds that can interfere with hormonal systems, is thought to participate in the development of thyroid dysfunction by influencing TH synthesis, transport, metabolism, and action [3,16,17,18]. Since the introduction of the term “endocrine disruptor” in 1999, EDCs, which include a variety of chemicals such as air pollutants, flame retardants, plasticizers, pesticides, and solvents, have been recognized as one of the most serious threats to human health [17,19]. Plasticizers are polymer additives commonly used to provide flexibility to a myriad of plastic materials, including children’s toys, cosmetics, construction materials, food contact materials (FCMs), and medical devices [20,21]. Due to the non-covalent bond between plasticizers and the plastic matrix, these substances can often diffuse out of the material and spread into the environmental matrices, thereby accumulating in ecosystems and entering the food chain [20,21]. Phthalates, a class of diesters of 1,2-phthalic acid developed in the last century to make polyvinyl chloride (PVC) more flexible and durable, have been shown to exert multiple toxic effects, including thyroid disruption in both animals and humans [3,20,22,23,24]. Given the critical role of EDCs in utero and early infancy, pregnant women and newborns are among the population groups most vulnerable to phthalate exposure [17]. Global regulation of phthalates has led to the introduction of alternative plasticizers, generally called “non-phthalate plasticizers” (NPP), which, while expected to be safer for the environment and human health compared to phthalates, can induce toxicity in various organs and can be associated with endocrine/reproductive dysfunction, although their effects on the thyroid are currently scarcely explored [24,25].

It is well known that the major sources of phthalates’ vehiculation into the human body are ingestion, inhalation, dermal or iatrogenic exposure, and, among these, the ingestion of contaminated food plays an important role in the whole process. Indeed, phthalates are somewhat abundant in many beverages, including mineral water, soft drinks, and wine, but also in dressings like olive oil or in ready meals [26], which are also among the main dietary sources of their counterpart, the NPP [26], later introduced into the economic sector due to their apparent safety and, above all, thanks to more flexible regulations on their use.

Furthermore, although the use of traditional plastic FCMs appears to be increasingly widespread worldwide, literature results on the migration of chemicals from food containers to food and/or the environment are still rather inconsistent, with values often scattered over a wide concentration range. These differences may be explained by the variety of exposure conditions employed experimentally, making it difficult to draw realistic conclusions on risk characterization and thus assess the health risks to consumers arising from migration [27].

This review aimed to comprehensively discuss the updated evidence on the role of phthalates and NPP on thyroid dysfunction and disease and the biological processes underlying this relationship, underscoring issues to be further investigated and possible strategies to be implemented to reduce the impact of these hazardous compounds on the thyroid and, in general, on human health. In particular, we will focus on the food industry, one of the main players for their inclusion in the cycle of human health. In this context, the key factors influencing the migration of chemicals from FCMs to food were also explored, along with potential programs that can be adopted by both national and international regulatory bodies, as well as by citizens in household environments, to minimize the migration rate as much as possible.

## 2. Thyroid Axis: An Overview

THs, produced by the thyroid, which is the only gland in humans to secrete and store its hormones, are responsible for regulating a myriad of physiological functions, mainly related to development and metabolism [18,21]. T3 and T4 are synthesized from the amino acid tyrosine thanks to the activity of sodium-iodide symporter (NIS), located on the basolateral membrane of thyrocytes, which allows the active transport of iodide into the gland, and by thyroid peroxidase (TPO), which catalyzes the oxidation of iodide to iodine that in turn is incorporated into tyrosine residues of thyroglobulin molecules to form diiodothyrosine and monoiodothyrosine in the follicular lumen [16]. Together with dual oxidase, TPO also promotes the coupling of mono- and diiodotyrosine residues to produce THs [28]. The transport of most circulating THs is mediated by serum TH–binding proteins, i.e., primarily thyroxin-binding globulin (TBG, which binds approximately 80% of T3 and 75% of T4) and, to a lesser extent, the low-affinity proteins albumin in mammals and primarily transthyretin (TTR) in other vertebrates [29,30]. Approximately 0.3% of total T3 (TT3) and 0.03% of total T4 (TT4) circulate in the free or unbound state (i.e., free T3—fT3 and free T4—fT4) due to the noncovalent and rapidly reversible binding to plasma proteins [31]. Based on the free hormone hypothesis, only the free or unbound hormones may cross the cell membrane of target cells; therefore, the measurement of fT3 and fT4 represents a more useful index for assessing true hormonal state [32].

Thyroid function is under tight control of the hypothalamic-pituitary-thyroid (HPT) axis, which is responsible for metabolic homeostasis in response to changes in metabolism and external stimuli [16,33]. If the thyroid acts to maintain constant basal levels of THs and subsequently keep the metabolic setpoint of cells unaltered, low TH concentrations trigger the release of thyrotropin-releasing hormone (TRH) from the hypothalamus, which stimulates the secretion of thyroid-stimulating hormone (TSH) in the anterior pituitary, which in turn induces the synthesis of T3 and T4 in the thyroid gland [33,34]. Conversely, increased levels of circulating THs generate a negative feedback mechanism on the HPT axis, inhibiting TRH and TSH release [35]. Besides, peripheral tissues can autonomously modulate TH signal by regulating TH transporters, deiodinases, and TH receptors [34]. TH transporters, which currently include 16 different molecules belonging to five distinct protein families, mediate TH transfer across the plasma membrane, and their tissue distribution and expression pattern contribute to the regulation of TH status [36]. The thyroid gland produces only 20% of T3, which is mainly generated through 5′ deiodination in peripheral tissues [28]. The differential expression of iodothyronine deiodinases (DIO1, DIO2, and DIO3), which belong to the thioredoxin fold superfamily, represents a further checkpoint in the control of serum and tissue TH concentration [34,35]. Indeed, while DIO1 (expressed in the thyroid, kidneys, and liver) and DIO2 (expressed in the thyroid, brown adipose tissue, central nervous system, and skeletal muscle) behave as “outer-ring” deiodinases by removing one iodine to convert T4 to T3, DIO3 inactivates both T4 and T3, promoting the conversion of T4 to reverse T3 and 3,5-diiodothyronine and of T3 to 3,3′-diiodothyronine in the brain, placenta, and pancreas and, under pathological conditions, also in other tissues [34,35]. In addition to DIO3, which is the physiological inactivator of THs, sulfation and glucuronidation, processes that increase compound water solubility, facilitate the excretion of conjugated TH via the bile, urine, and gut [35,37]. Furthermore, T3 primarily exerts its effects by regulating target gene expression through binding to nuclear TH receptors (TRs), which belong to the superfamily of nuclear hormone receptors whose two major isoforms, TRα and TRβ, display tissue specificity and distinct developmental patterns and undergo post-translational modifications [16,38]. In particular, the splicing products TRα1 and TRβ1 are expressed in a broad range of organs and tissues, whereas TRβ2 expression is restricted to the hypothalamus, pituitary, inner ear, and retina [39]. On the other hand, TRα2, which is unable to interact with T3 and thus reduces T3 action, is abundant in neural tissues [16,38].

Upon T3 binding, the TR forms a homodimer or a heterodimer with retinoid X receptor and binds to DNA regulatory regions of target genes, known as thyroid response elements, stimulating or inhibiting gene transcription by modifications of chromatin remodeling complexes [16,40]. The genomic effects of THs are made possible by a switch between co-repressor and co-activator proteins [35]. In fact, while co-repressors bind to unbound TR, thus promoting gene repression, binding of T3 to the TR enables the co-repressor release and the recruitment of the co-activator, which in turn recruits enzymes that acetylate histones, with subsequent activation of transcription [16,35]. Alternatively, non-genomic actions of THs, namely extra-nuclear pathways, involve TH receptors on the plasma membrane that trigger signal transduction often mediated by secondary messengers and may eventually culminate in transcriptional activation of target genes [35,41].

**Figure 1:**
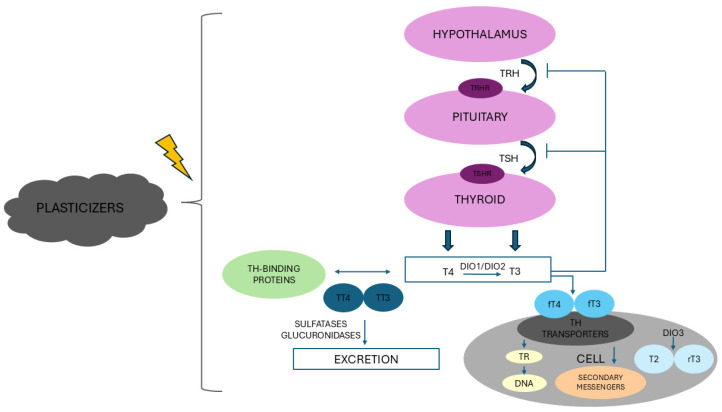
Schematic representation of checkpoints within the hypothalamic-pituitary-thyroid axis on which plasticizers may exert their effects (see text for details). Abbreviations: DIO: deiodinase; fT3: free triiodothyronine; fT4: free thyroxine; rT3: reverse triiodothyronine; T2: diiodothyronine; T3: triiodothyronine; T4: thyroxine; TH: thyroid hormone; TR: thyroid receptor; TRH: thyrotropin-releasing hormone; TRHR: thyrotropin-releasing hormone; TSH: thyroid stimulating hormone; TSHR: thyroid stimulating hormone; TT3: total triiodothyronine; TT4: total thyroxine.

In light of the complexity of the thyroid axis and the multiple levels of control of mechanisms of action of THs, it is not surprising that the thyroid gland is particularly sensitive to environmental factors, which may cause thyroid dysfunction, as well as TC and other disorders [42]. Thyroid disruptors, a subfamily of EDCs, are widely diffused in the environment and in daily-use products and may affect thyroid function at any level of this regulation by interfering with any aspect of the HPT axis, mimicking and/or disrupting the synthesis, release, transport, action, and metabolism of THs on target tissues [43,44,45,46,47] (Figure 1).

In the following sections, we will therefore focus on the thyroid-disrupting actions of plasticizers resulting from epidemiological studies and the biological process that might explain this relationship.

## 3. Plasticizers

### 3.1. Phthalates

**Figure 2:**
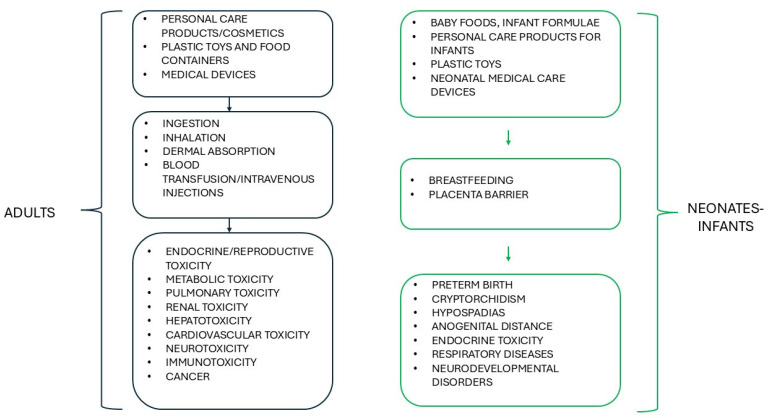
Diagram illustrating the sources and routes of exposure to phthalates and their effects on health based on the developmental stage (see text for details).

In 2022, the global production of plasticizers reached approximately 11 million tons, and the market volume of this class of compounds is estimated to continue to grow to exceed 14.3 million tons in 2030 [48]. Phthalates, which, beyond being used as plasticizers in PVC, polyethylene, polyethylene terephthalate, and polyvinyl acetate to enhance transparency, strength, plasticity, and durability of plastic materials, can also be found in adhesives, cosmetics, fertilizers, food containers, lubricants, medical devices, paints, pesticides, and toys, are characterized by an estimated worldwide production of between 6 and 8 million tons per year [49,50]. They are colorless, synthetic organic compounds resulting from the esterification of phthalic acid, generally showing low melting and high boiling points and low water solubility [50,51]. Over 25 different types of phthalates (di-(2-ethylhexyl) phthalate—DEHP; dibutyl phthalate—DBP; diethyl phthalate—DEP; di-isononyl phthalate—DiNP; di-isodecyl phthalate—DiDP; benzyl butyl phthalate—BBP; and dimethyl phthalate—DMP; the most common) are employed in commercial practices, and due to their widespread use in both domestic and industrial settings and the lack of covalent bond to the polymer, they can diffuse from these products through evaporation, leaching, and abrasion, and, as such, are ubiquitously detectable in all environmental matrices, i.e., air, water, soil, and food [49,50,51]. Although phthalates do not generally persist in the environment like other organic compounds, their photodegradation and hydrolyzation rates are relatively slow under natural conditions, and half-lives of phthalates can range from 3 years for DMP to 2000 years for DEHP [52,53]. Exposure routes to phthalates include inhalation by subjects living near phthalate manufacturing industries, dermal absorption through the daily use of plastic packages of personal care products, and ingestion of food items, the latter considered the main source of exposure, with fatty foods, such as milk, butter, and meat, as the primary contributors [52,54]. In addition, fetuses and infants can be exposed to phthalates via breastfeeding and the placenta, thanks to the ability of these chemicals to be transferred to breast milk and cross the placenta-blood barrier [52]. Exposure to phthalates in neonates and infants may also occur through infant formulae and baby foods, skin care products, and plastic toys [54,55]. Phthalates are also contained in medical devices composed of PVC; therefore, certain medical procedures, such as blood transfusion, parenteral infusion of drugs, and, with regard to neonatal intensive care units, contact with feeding tubes and endotracheal tubes, may represent a further relevant route of exposure [53]. Phthalates may potentially affect human health in multiple organs and systems, depending on the stage of development and sex [56,57,58] (Figure 2).

In humans, phthalates have a short half-life (approximately 5 h) and, upon absorption into cells, are firstly hydrolyzed and then conjugated to form the hydrophilic glucuronide conjugate [59,60]. Depending on the type of phthalate, excretion occurs through urine for short-branched phthalates or through urine and feces for long-branched phthalates, although some phthalates such as DEHP and its metabolites may also be excreted in sweat [59,60]. Phthalates, in addition to being considered EDCs, are supposed to potentially act as teratogenic, mutagenic, and carcinogenic agents even at low concentrations [53]. Indeed, DEHP, the most widely used phthalate with an estimated annual worldwide consumption of 3–4 million tons and accounting for half of the total plasticizer production, has also been classified as possibly carcinogenic to humans by the International Agency for Research on Cancer [61,62]. In the European Union, the use of DEHP, DBP, DEP, DiNP, DiDP, and dioctyl phthalate has been restricted in children’s toys since 1999, and this ban, which has become permanent, was also extended to children’s products in 2007 and to di-isobutyl phthalate (DiBP) in 2018 [63]. As being classified as toxic to reproduction, DEHP, DBP, DiBP, and BBP have been restricted in Europe since 2020 for use in various products, including children’s swimming aids, coated fabrics and paper, flooring, footwear, mattresses, office supplies, and recreational equipment [63]. Fourteen phthalates have been included in Annex XIV of the REACH Regulation of the European Union and Regulation 2021/2045, adding endocrine-disrupting properties to DEHP, BBP, DBP, and DiBP, and banning their use (individually or in combination) at concentrations greater than or equal to 0.1% by weight in FCMs and medical devices [64]. Furthermore, based on the updated risk assessment of the European Food Safety Authority, the tolerable daily intake (TDI) in FCMs for DBP, BBP, DEHP, and DiNP was set at 50 µg/kg body weight (bw) per day, while TDI for DiDP was established at 150 µg/kg bw per day [60]. The current exposure to these compounds in the European general population is much lower than TDIs (4–7 times for DBP, BBP, DEHP, and DiNP and 1500 times for DiDP) and does not raise public health concerns [65]. In the 2022 Final Rule, the U.S. Food and Drug Administration revoked the authorization for the food contact use of 23 phthalates, and currently only nine phthalates are authorized for use as FCMs [66]. Although restrictions in various parts of the world have reduced overall human exposure to these substances, the detection rates of urinary phthalate metabolites still exceed 90% in several populations, and considering that phthalates have short half-lives in humans, these elevated levels are the result of recent exposure, confirming their current ubiquitous presence in the environment [66,67,68,69,70].

### 3.2. Non-Phthalate Plasticizers

Because phthalates have been linked to a wide range of health outcomes that have prompted governmental and non-governmental organizations to strictly regulate their use, alternative plasticizers such as tributyl acetyl citrate (ATBC), di(2-ethylhexyl) adipate (DEHA), diisononyl 1,2-cyclohexanedicarboxylic acid (DINCH), di(2-ethylhexyl) terephthalate (DEHTP), diisononyl adipate, and epoxidized soybean oil have been introduced in recent years to replace phthalates, especially in sectors such as packaging, medical devices, and automotive [25,71,72,73]. Global production of NPP was estimated at 3.31 billion USD in 2023 and is expected to increase at a compound annual growth rate of 4.25% over the period 2024–2030 [68]. In Europe, DEHA and DINCH are the most widely used plasticizers, with an annual production of over 10,000 tons [74,75]. Like phthalates, NPP are not chemically bound to their source material and therefore may enter all the environmental matrices (water, soil, sediments), biota, and food items via abrasion, evaporation, leaching, and/or migration [21,76]. Of interest, high levels of NPP have been measured in domestic dust as they migrate from children’s toys, childcare products, and school supplies [76]. Nonetheless, exposure to NPP such as DEHP and DINCH mainly occurs via the oral route, contributing to 90% of internal exposure, due to their presence in FCMs in numerous world areas such as the United States and Europe [77,78]. As demonstrated by a survey examining phthalate and NPP concentrations in foods and food handling gloves from U.S. fast food restaurants, although di-isononyl phthalate (DnBP) and DEHP were the most frequent phthalates in foods (81% and 70%, respectively), DEHA and DINCH were detected in 41% and 14% of foods, respectively [74]. Storage time and temperature, as well as the fat content of food items, appear as among the most relevant factors in controlling the amount of plasticizers migrating into packaging foods [79]. Indeed, a recent study reported high levels of DEHA in dairy products stored at 4 °C for 30 days with an order of migration (butter > cheese > milk) that was consistent with fat contents of products and the lipophilic nature of DEHA [80]. For DEHA, the European Union Scientific Committee on Food has established a TDI of 0.3 mg/kg bw and a specific migration limit of 18 mg/kg food [81], while a TDI of 1 mg/kg bw has been set for DINCH and DEHTP based on renal effects and combined toxicity/carcinogenicity, respectively [82,83].

Although considered safe, phthalate substitutes may potentially pose a threat to human health, as shown in experimental models, where DEHA exhibited hepatotoxicity, neurotoxicity, and cardiotoxicity, while DINCH exposure affected reproductive function, liver metabolic capacity, and induced cellular stress and inflammation [80,84,85,86,87]. Once entered into the human body, NPP are rapidly metabolized into their respective hydrolytic/oxidative monoesters and excreted in urine after partial glucuronidation [88]. Interestingly, metabolites of DEHPT and DINCH were found in 95.6% and 92.2% of 7-year-old Japanese children, with a significant 5-fold increase in DEHTP metabolites and a 2-fold increase in DINCH metabolites over a 5-year period, although the measured values were much lower than the health-based guidance and TDIs [84]. Two DEHTP metabolites were detectable in over 95% of the urinary specimens of a representative North American population, including children aged 3 to 5 years, suggesting diffuse exposure to phthalate substitutes [89]. In agreement with these findings, a marked increase in daily excretion rate of DINCH and DEHTP (by ~10–68% and ~100%, respectively) between 2009 and 2017 was also observed in two cohorts of German and Danish young adults, indicating that the increasing use of NPP in industrial applications requires careful monitoring of exposure, especially in certain world areas (Asia, Africa, Oceania) where data on human biomonitoring are still scarce [70,90].

## 4. The Association Between Exposure to Phthalates and Thyroid Dysfunction

So far, numerous studies have explored the relationship of exposure to phthalates and effects on the thyroid gland in humans in different group populations, especially pregnant women, although the direction and strength of these associations remain currently somewhat inconsistent (Table 1). The meta-analysis by Kim et al. [91], including thirteen studies published in the years 2007–2017 for a total of 12,674 patients, first investigated the association between DEHP exposure and HPT function, evaluating the urinary concentrations of three of its metabolites in relation to circulating TH levels. Overall, monoethylhexyl phthalate (MEHP) was inversely associated with TT4, although this correlation was observed only in pregnant women, but not in adults and children in the subgroup analysis [91]. In addition, MEHP did not show any correlation with fT4 and TSH [91]. Levels of mono (2-ethyl-5-hydroxyhexyl) phthalate (MEHHP) in urine were negatively associated with TT4, although children exhibited a positive correlation of MEHHP with both TT4 and TSH [91]. Similar results were reported for urinary concentration of mono (2-ethyl-5-oxohexyl) phthalate (MEOHP) that in children was positively correlated with TT4 and TSH levels, while in the whole sample it was modestly correlated with increased TSH [91]. These differences by subgroups can be attributed to the timing of sample collection in pregnant women as thyroid function fluctuates with gestational age or to the longer duration of exposure in children than in adults [91]. Therefore, despite certain limitations of some of the studies included, namely the cross-sectional design, the lack of correction by urinary creatinine levels or specific gravity, and the single measurement of DEHP metabolites (even though it is supposed to represent long-term exposure), these findings suggest that DEHP may interfere with thyroid function by increasing TSH and reducing TT4 levels [91]. On the other hand, it should be noted that phthalates, like all EDCs, are characterized by U-shaped dose–response curves, and analytical methods may not detect a non-linear association between DEHP exposure and thyroid effects [91]. In a prior prospective pregnancy birth cohort study aimed at evaluating the impact of phthalate mixtures (reflecting exposure to six parent phthalates for a total of nine urinary monoester metabolites) on THs and including 202 American pregnant women and 276 newborns enrolled between 2003 and 2006, the authors reported a decrease in maternal serum TT4 for each 10-fold increased concentration of urinary monoethyl phthalate (MEP), the only DEP metabolite, measured at 16 weeks of pregnancy [92]. Among newborns, the average increase in urinary maternal monobenzyl phthalate (MBzP, a metabolite of benzyl butyl phthalate—BBzP) during pregnancy (calculated from maternal urinary samples collected at approximately 16 and 26 weeks’ gestation) was negatively associated with TSH in cord serum, confirming a central effect of phthalates on the HPT axis [92]. Interestingly, an increase in the phthalate index, which incorporates combined concentrations of individual phthalate metabolites to assess the relative contribution of mixture components in the overall association with THs, was inversely associated with maternal TT4, being MEP and mono-3-carboxylpropyl phthalate (MCPP, a DBP metabolite) the major contributors, and with cord TT4 and TSH, being MBzP and mono-isobutyl phthalate (MiBP, a DiBP metabolite) the primary drivers in this decrease [92]. Furthermore, while child sex appears to modify the effects of maternal MiBP on TH levels of newborns, promoting a decrease in cord serum TT4 and fT4 in males, but not in females, in a subset of women (77%) for whom urinary iodine measurements were performed, the overall results for most associations remained unchanged when maternal urinary iodine was added as a covariate in the adjusted multivariable model [92]. If this study underscores the possibility of different effects of phthalates on male and female infants and the importance of assessing co-exposure to phthalate mixtures, it also suggests that weighted quantile sum regression, used to create a phthalate index, may not be ideal for studying non-monotonic exposure-outcome associations as well as exposures potentially characterized by synergy or antagonism between chemicals [92]. A subsequent prospective birth cohort study evaluating a total of fifteen phthalate metabolites on 677 Puerto Rican women in the years 2012–2017, who provided blood and urine samples at two time points during pregnancy, reported significantly positive associations of TT3 with most metabolites but, unlike Romano et al. [92], did not observe any significant association between urinary MEP concentration and TT4, probably due to higher levels of exposure and measurements conducted at different median gestational ages (16–20 weeks vs. 10–23), suggesting differential effects of phthalates on THs depending on the stage of pregnancy [93]. While no associations were reported between phthalate exposure and TSH, monocarboxyoctyl phthalate (MCOP, a metabolite of DiNP) and MCPP were significantly correlated with higher serum levels of FT4 and TT4 [93]. Within a Norwegian prospective population-based pregnancy cohort study, Villanger et al. [94] evaluated the relationship between levels of twelve urinary phthalate metabolites and six biomarkers of thyroid function (measured at approximately 17 weeks’ gestation) on 1072 selected pregnant women (534 mothers of children with diagnosis or symptoms of attention-deficit hyperactivity disorder—ADHD and 538 mothers of controls recruited from all of Norway between 2003 and 2008) and whether these associations can be modulated by dietary iodine intake, assessed by a food frequency questionnaire completed by participants at 22 weeks of gestation. In particular, the authors separately estimated the effects of factor 1, including high loadings of mono-n-butyl phthalate (MnBP), MBzP, and MiBP, deriving from low-molecular-weight phthalates, which are principally used in dietary supplements, medical devices, and personal care products, and factor 2, mainly explained by high loadings of DiNP and DEHP metabolites, originating from high-molecular-weight compounds that are employed in PVC products and frequently detected in food items [94]. Factor 1 was significantly associated with increased levels of TT3 and fT3 index (estimates based on T3 uptake), with these associations appearing to be slightly stronger in women with low iodine intake (below 150 µg per day), while factor 2 was significantly inversely associated with TT3 and fT3 index, without any effect modification by iodine intake [94]. Of note, unlike what was reported by [92], iodine intake significantly modified the negative associations of factor 1 with the fT4 index and TT4 and the positive association with TSH among women with an iodine intake >150 µg per day. Overall, if these results support findings from experimental studies showing that NIS in the thyroid may represent a target of several phthalates (see Section 4.2), which can then enhance the thyroid autoregulatory response to low iodine intake by inducing an increase in T3, conversely, iodine status may influence phthalate-thyroid function relationships across different populations of pregnant women [94]. Consistent with [93], this study also found no evidence of association between urinary MEP concentration and significant changes in biomarkers of thyroid function or effect modification by iodine intake during pregnancy [94]. The use of data from mothers of children with ADHD, which may have caused residual bias from factors strongly associated with this neurodevelopmental disorder, and the lack of adjustment for maternal body mass index (BMI), which can be related to both phthalate exposure and thyroid function, represent the two major limitations of this study [94,95,96]. In a subpopulation of Korean adults (n = 1254, aged ≥19 years) participating in a national survey conducted from 2015 to 2017 and aimed at exploring the exposure levels to environmental pollutants, Choi et al. [92] demonstrated that, among the eight phthalate metabolites considered, all (including MnBP and MBzP) were significantly and positively correlated with TT3, as previously reported by [95], while none of them was significantly associated with TSH. Except for MnBP and MCPP, all chemicals were also significantly associated with decreased levels of fT3, while MBzP, MCPP, mono-(2-ethylpentyl-5-carboxy) phthalate (MECPP, a DEHP metabolite), and MCOP showed an inverse association with TT4 [97]. All metabolites and DEHP metabolites were also associated with increased levels of peripheral DIO activity and TBG, respectively, findings corroborated by the positive correlation with TT3, and this may represent a potential mechanism of thyroid disruption by phthalates [97]. No association was observed between any chemical and TPO and thyroglobulin (TG) autoantibodies; however, the presence of autoimmunity appears to modulate certain relationships between phthalate exposure and thyroid status, modifying the susceptibility to chemical exposure, although the small number of subjects positive for TPO and/or TG antibodies may have resulted in low statistical power [97]. Indeed, while none of the phthalate metabolites displayed significant associations with fT3 in the presence of thyroid autoantibodies, in patients with no thyroid autoimmunity, all metabolites, except for MnBP, were inversely associated with fT3 [97]. Additionally, differences in the strength of association by autoimmunity status were also observed for fT4 and TBG [97]. When regression analysis was performed evaluating the association between multiple exposures to phthalates (i.e., molar sum of urinary DEHP metabolites MnBP and MBzP, and molar sum of MCOP, MCPP, and monocarboxy-isononyl phthalate—MCNP, the latter a metabolite of di-isodecyl phthalate) and effects on the thyroid, the authors reported comparable results with those from the single-chemical model [97]. A Swedish study embedded in a population-based prospective pregnancy cohort and comprising 1996 women with a median gestational age of 10 weeks (period of recruitment September 2007–March 2010) evaluated the association between the levels of fourteen phthalate metabolites and a broad set of thyroid parameters [98]. The molar sum of DEHP metabolites was significantly inversely associated with fT4, fT3 (non-linear relationship), and TT3 concentrations, in line with [89], and with an increase in TSH/fT4 ratio, indicating an effect of DEHP on the HPT axis, which supports data from experimental studies (see Section 4.2) [98]. Increased levels of DiNP metabolites (molar sum) were significantly negatively associated with TT4 levels and, consequently, with a reduced TT4/TT3 ratio [98]. MEP was poorly correlated with thyroid function, except for a negative association with fT4, thus confirming previous findings [98]. Conversely, higher concentrations of DBP and BBzP were associated with increased levels of TT3, lower fT4/fT3 and TT4/TT3 ratios, and higher TT4/fT4 and TT3/fT3 ratios, which overall are suggestive of enhanced T4 metabolism and an increase in levels of thyroid hormone binding protein [98]. Notably, none of the phthalate metabolites analyzed exhibited significant effects on TSH levels or thyroid autoantibodies [98]. Furthermore, the associations between individual metabolites and various outcomes reflected the same direction as the associations observed for the correspondent parent phthalate, which, while supporting the greater validity of the molar sum of phthalate metabolites as a proxy for phthalate exposure and ruling out that the separate associations are due to chance, the calculation of total exposure does not account for possible differences in the rate of metabolism or binding affinities of individual metabolites [98]. Yang and coauthors measured serum levels of thyroid parameters and urinary concentrations of ten phthalates in early pregnancy in 42 Chinese women with a diagnosis of subclinical hypothyroidism (SCH) and 84 euthyroid women matched for age and BMI enrolled between August 2019 and January 2020 [99]. The molar sum of DEHP metabolites, MECPP and MEOHP (the latter two are DEHP metabolites), was significantly positively associated with TSH, as shown in [91], and with the variable fT4 × TSH, which reflects the thyroid’s physiological negative feedback and is comparable to the TSH/fT4 ratio, evaluated by [93], supporting the hypothesis that phthalates may interfere with the HPT axis at different setpoints (see Section 4.2) [97]. In contrast, no associations were observed between single metabolites, summary variables, fT4, and TPO antibodies [99]. In the evaluation of the relationship between quintile levels of total phthalate exposure and serum concentration of thyroid function biomarkers, despite the absence of significant differences, there was evidence of upward trends for TSH, fT4 × TSH, and TPO antibodies, and a downward trend for fT4 [99]. Of note, while the small sample size may have reduced the statistical power of some associations, the cross-sectional design does not allow us to infer the directionality of causality [99]. Also, the selection of pregnant women with SCH could have caused inaccuracy in measurement estimates as thyroid function is subject to dramatic changes during pregnancy [99]. Using 2007–2008 data from the U.S. National Health and Nutrition Examination Survey, Xiang et al. [3] analyzed the association between phthalate exposure (for a total of eleven metabolites) and thyroid function in 356 adolescents aged 12–19 years. The study reported a positive correlation of the molar sum of DEHP metabolites with TT3 and TT4 and of MCOP with TT3 and TSH, whereas MCPP was inversely correlated with TT3 and TT4 and MCNP with TSH [3]. Importantly, TT3 concentration significantly increased with increasing levels of phthalate metabolite mixtures, indicating a strong relationship between mixed exposure to phthalates and thyroid health in adolescents [3]. A few years before, in a prospective follow-up study, Huang et al. [100] recruited 166 Taiwanese children (aged 2–18 years) who had suffered the phthalate food scandal in 2011 (some phthalates had been illegally added to foodstuffs and medications) in three visits from 2012 to 2016, estimating their daily phthalate intake through the administration of a questionnaire and the relationship between phthalate exposure (nine metabolites measured on three urine specimens, one in each visit) and serum TH levels. The median urinary levels of the molar sum of DBP metabolites and monomethyl phthalate (MMP) were significantly higher at visits 2 and 3 than at visit 1, and the estimated daily intake of DiBP and DnBP at visit 2 was significantly higher than that at visit 1 [100]. Besides, after adjusting for covariates, urinary MMP was significantly inversely associated with TT3, TT4, and fT4, while the molar sum of DEHP metabolites, MEHOP, and MEHPP were positively associated with fT4 [100]. Overall, these findings support a long-term disruptive effect of phthalate exposure, particularly to low-molecular-weight metabolites such as MMP, the major sources of which for humans are cosmetics and personal care products, on thyroid function homeostasis, although the relatively small number of participants and loss of subjects during follow-up may have overestimated the observed effects [100]. Finally, a recent prospective cohort study performed in the years 2019–2022 and including a total of 672 pregnant women up to 13 weeks of gestational age who provided a blood sample and two urine samples in each trimester evaluated the effects of different levels of exposure to phthalate metabolites (a total of seven compounds) on TH levels throughout pregnancy [101]. In particular, fT4 levels significantly increased (from 2 to 3.7%) with increasing quartile exposure to MECPP and the molar sum of DEHP, DBP, and all seven low- and high-molecular-weight metabolites, while increased TSH levels (5–16%) were observed for all single metabolites (except for MEHHP) and their molar sum [101]. An increasing (2.2%) and a decreasing (−2.7%) trend of serum TT3 were detected across the quartiles of MEP and in correspondence with the fourth quartile of the molar sum of DBP metabolites, respectively [101]. Furthermore, a higher TSH/fT4 ratio (range 8.7–22.2%) was associated with the highest quartiles of almost all individual (MEP, MiBP, MnBP) or molar sums of all low-molecular-weight and DEP metabolites [101]. These hormonal alterations, which, except for fT4, are more pronounced in the second and third trimesters, are suggestive of different disrupting effects of phthalates on thyroid function depending on the timing of exposure [101]. However, while stratification of the cohort into three trimesters resulted in smaller subgroups for each trimester and, consequently, potentially less precise estimates, the lack of assessment of the combined effects of phthalate metabolites prevents estimating the full impact of phthalate exposure on the thyroid [92,101].

In summary, despite the large amount of data produced in the evaluation of the relationship between phthalate exposure and thyroid function, results remain inconclusive due to the great heterogeneity of the study designs, the populations evaluated, the type and number of phthalate metabolites analyzed, the timing (in pregnant women), and the method (single-spot or multiple specimens) of measurement of compounds across studies. Nonetheless, some considerations can be drawn: in pregnant women, most phthalate metabolites or their parent diester phthalates appear to reduce TT4 levels and concomitantly increase TT3, probably through a common pathway involving an influence on deiodinase activity (which indeed shows a positive correlation with some phthalate metabolites). Additionally, the reported reduction of TT4/fT4 and TT3/fT3 ratios in relation to phthalate exposure indicates interference with TH-binding proteins. The positive association observed between certain compounds and TSH can instead be explained by the negative physiological feedback of the thyroid gland on the HTP axis in order to maintain TH homeostasis. As for children and adolescents, the results are conflicting, with reported positive or negative associations of urinary phthalate metabolites with TT3, TT4, and fT4. In addition to the limited evidence, certain variables such as iodine intake and the presence of thyroid autoimmunity could act as modifiers in the relationship between phthalate exposure and thyroid parameters (Table 1).

### 4.1. The Association Between Phthalate Exposure and Thyroid Disease

A limited number of studies have also investigated the association between phthalate exposure and thyroid disease (Table 2). Within a case-control study carried out between August 2014 and March 2015, Sur et al. [102] measured plasma DEHP and MEHP in 29 Turkish children diagnosed with Hashimoto’s thyroiditis (HT), a disorder characterized by a gland enlarged with distinctive ultrasound pathological features and elevated levels of TPO antibodies, and 29 controls age-matched with cases and without any chronic, autoimmune, or genetic disease. The authors found a non-significant 21.5% increase in DEHP levels in the HT group compared to controls, while the 138.2% increase in MEHP levels in cases compared to the control group was statistically significant (*p* < 0.01) [102]. Besides, while no significant associations were reported between DEHP or MEHP and thyroid parameters (fT4, fT3, TSH, TPO antibodies), MEHP was positively associated, although with a borderline correlation, with levels of selenoprotein P, which, produced in the liver, is the major plasma selenium (Se) transporter to tissues and considered a surrogate biomarker of Se body status [102,103,104]. Importantly, deficiency of Se, a trace element essential for TH synthesis and metabolism, may affect the physiological thyroid function [103]. Conversely, selenoproteins, which contain Se in their active site, are potent antioxidants capable of eliciting reactive oxygen species (ROS) and thus mitigating oxidative stress [103]. In the research by Sur et al. [102], MEHP appears to consistently interfere with Se and selenoproteins such as glutathione peroxidase (GPx) 1, GPx4, and thioredoxin reductase, as reported in prior experimental studies [105,106].

A Chinese pilot case-control study of pregnant women (average gestational age of 7.3 weeks) including 42 women with SCH, a condition characterized biochemically by serum TSH levels above the upper reference limit in the presence of normal fT4 levels [107]), and 84 controls matched with cases by age and BMI, investigated the relationship between phthalate exposure and SCH during early pregnancy [99]. Among the ten phthalate metabolites analyzed, only MEP presented a significantly higher concentration in SCH women compared to controls, probably due to the elevated use of DEP, the parent metabolite of MEP, in personal care products [99]. Notably, the molar sums of DEHP metabolites, MEP, MECPP, and MEOHP were significantly associated with increased risk of SCH (Odds Ratios—ORs of 1.92 [95% Confidence Interval—95%CI: 1.01–3.78]; 1.42 [1.03–1.95]; 1.89 [1.01–3.60]; 1.81 [1.02–3.22]; respectively, all adjusted for covariates), suggesting that DEHP is a highly relevant risk factor for SCH as it is largely employed in a multitude of consumer products and its metabolites are among the predominant compounds detected in the majority of biomonitoring studies [99,108].

A recent case-control study [109] conducted in the first four months of 2019 on 20 Turkish adolescents (ages 12–15 years) diagnosed with thyroid colloid cyst (TCC), a common benign thyroid nodule of low clinical significance and containing a dense fluid consisting of a concentrated solution of TG [110], and 22 controls with normal neck ultrasonography and age-matched with cases, explored differences in the levels of fourteen phthalate metabolites between the two groups. Significantly higher levels of the high-molecular-weight phthalate metabolites MEHP, MEHHP, and monocarboxy isononyl phthalate (MCiNP) were found in cases than in healthy adolescents, while the urinary concentration of the low-molecular-weight phthalate metabolites MiBP and MMP was significantly more elevated in the control group [109]. After grouping phthalate metabolite levels into tertiles, TCC adolescents in the highest tertile of MCiNP, MCPP, and MiBP had lower adjusted ORs than those in the first tertile (OR = 0.07, *p* = 0.005; OR = 0.015, *p* = 0.049; OR = 0.08, *p* = 0.029, respectively), and this seemingly surprising result might be based on the sex-, dose-, and metabolite-dependent effects of phthalates on the thyroid [73,109]. Conversely, TCC adolescents in the highest tertile of MEHP had a 14.4-fold higher OR (*p* = 0.009) than controls, confirming the toxic effects of DEHP and its correlation with thyroid dysfunction, as previously seen for SCH [99]. However, it should be noted that the pathological significance of TCC is currently unknown, while the small sample size, the single-spot urine measurements, and the lack of assessment of associations between phthalate exposure and thyroid parameters represent relevant limitations of the study [109].

Three studies have also evaluated the potential role of phthalates in increasing the risk of TC development. In a multicenter, cross-sectional study, Marotta and co-authors [111] enrolled a total of 55 Italian subjects, 27 of whom were affected by thyroid nodules and 28 diagnosed with differentiated thyroid cancer (DTC, the most common endocrine malignant tumor arising from thyroid follicular cells, [112]), and measured DEHP and MEHP on a blood sample of each patient. Even in multivariate analyses, DEHP exposure was related to a 15-fold higher risk of DTC in patients with thyroid nodules (OR = 15.97, 95%CI: 1.59–142.13, *p* = 0.018) [111]. However, no significant differences in DEHP levels were found in DTC patients compared to thyroid nodule subjects, nor was DEHP significantly correlated with TSH levels (TSH is an established predictor of malignancy in thyroid nodules), thus demonstrating that DEHP may act as an independent risk factor of DTC in a dose- and TSH-independent manner, probably by exerting a direct mutagenic action on thyroid cells [111,113]. A subsequent study conducted in China during March-December 2016 including sex-matched individuals with histologically confirmed TC (n = 144), benign thyroid nodules (n = 138), and healthy subjects (n = 144) recruited from the same hospital as the two first groups evaluated the risk of TC and benign nodules in relation to phthalate exposure [114]. Among the eight urinary phthalate metabolites, six of them (monobutyl phthalate—MBP; MEP; MBzP; MEHP; MEOHP; and MEHHP) were detectable in more than 90% of patients, and the highest tertiles of MMP, MEHHP, and MEHP were significantly associated with increased risk of TC (OR = 2.49, *p* trend = 0.001, OR = 2–18, *p*-trend = 0.008, OR = 1.74, *p*-trend = 0.04, respectively), while the second (OR = 0.25, 95%CI: 0.13–0.48) and third tertiles (OR = 0.20, 95%CI: 0.10–0.40) of MBP were significantly inversely associated with the occurrence of TC compared to the lowest tertile (*p*-trend < 0.001) after adjusting for covariates [114]. These relationships were also confirmed when evaluating phthalate exposure as a continuous variable, with adjusted OR = 1.11 (95%CI: 1.01–1.22), OR = 1.53 (95%CI: 1.19–1.96), OR = 1.46 (95%CI: 1.09–1.91), and OR = 0.45 (95%CI: 0.34–0.60) estimated for logarithmic-transformed concentrations of MMP, MEHHP, MEHP, and MBP, respectively [114]. For benign nodules, the authors reported similar estimates in terms of magnitude and effect, with MMP, MEOHP, MEHHP, and MEHP showing significant and positive correlations in both categorical and continuous analyses, and MBP being negatively associated [114]. Of note, a significant effect modification by sex was found between MEHOP exposure and the risk of thyroid malignancy (*p* = 0.01) and for MEP in relation to the occurrence of thyroid benign nodules (*p* = 0.03), with males having a higher risk than females in both conditions, probably due to the disruption of sex hormones by the two phthalate metabolites, although the small number of males requires caution in interpreting this result [114]. Finally, a 1:1 pair-matched case-control study [115] involving 111 patients with newly diagnosed papillary thyroid cancer (PTC) (the most common histotype of DTC whose prevalence accounts for more than 90% of cases [116,117]), and an equivalent number of non-PTC subjects recruited in the same hospital and during the same study period (June–September 2017), reported that PTC subjects had a significantly higher concentration of urinary DEHP metabolites, although the moderate and strong correlation coefficients between DEHP metabolites and between DEHP metabolites and the other metabolites analyzed (six compounds in total) suggests that PTC subjects could not be more exposed to DEHP. Additionally, both the molar sum of DEHP metabolites (OR = 3.51, 95%CI: 1.64–7.49, *p* = 0.001) and single DEHP metabolites (i.e., MEHP, MEOHP, MECPP, and MEHHP) were all significantly associated with an increased risk of PTC (ORs varying from 2.07 to 7.30) in the multivariable model, and the molar sum of DEHP metabolites remained positively associated with PTC after adjusting for covariates and other phthalate metabolites (OR = 5.35, 95%CI: 1.61–17.83, *p* = 0.006) [115]. As also documented by Liu et al. [114], most metabolites were characterized by dose-response non-monotonic relationships with PTC [115]. Notably, iodine status appears to influence the relationship between certain phthalate metabolites and risk of PTC. Indeed, while DEHP metabolites showed a significantly positive association with PTC incidence regardless of low or high urinary iodine levels, MBP was significantly associated with an increased risk of PTC only in the presence of urinary iodine concentration < 247 µg/L, indicating that an adequate iodine intake may counteract the thyrotoxicity of certain phthalates [115].

Overall, the current epidemiological evidence, despite being overall scarce, is suggestive of multiple toxic effects of phthalates on the thyroid, with the possibility of the development of milder diseases such as HT and SCH, up to TC. In particular, although radiation exposure is the only factor strongly associated with the development of TC, accounting for one third of cases without the existence of a safe threshold dose [118,119], the accumulated knowledge indicates that several phthalate metabolites exhibit marked endocrine-disrupting properties and thus may also contribute to increasing the risk of both thyroid benign nodules and thyroid malignancy, with a potentially different action by sex and iodine status. On the other hand, given the cross-sectional design and the relatively low number of patients involved in these studies, which may give rise to imprecise estimates and cannot demonstrate a causal relationship due to the simultaneity of exposure and outcome assessment, larger prospective studies based on multicenter case recruitment points would be desirable to confirm these relevant preliminary findings. Furthermore, a more comprehensive evaluation of the effects of phthalates should include measuring exposure to other EDCs, as well as nutrient intake and sex hormone levels.

**Table 2:** Clues and pitfalls in the relationship between phthalate exposure and thyroid diseases.

Clues	Reference	Pitfalls	Reference
Plasma MEHP levels significantly and markedly increased in children with HT compared to controls	[102]	No significant changes in plasma MEHP levels between HT and healthy children	[102]
Plasma MEHP levels mildly correlated with plasma concentration of selenoprotein P	[102]	No associations revealed between plasma DEHP/MEHP levels and thyroid parameters (fT4, fT3, TSH, TPO antibodies)	[102]
Urinary levels of MECPP, MEP, MEHOP, and molar sum of DEHP metabolites significantly associated with a higher risk of SCH during pregnancy	[99]	Small sample size	[99,102,109,111,114,115]
Urinary concentrations of MEHP, MEHHP, and MCiNP significantly higher in adolescents with TCC than in the control group	[109]	No significant differences in urinary levels of phthalate metabolites between pregnant women with SCH and controls except for MEP	[99]
Higher ORs for adolescents with TCC in the highest tertile of MEHP compared to controls	[109]	Urinary concentration of MiBP and MMP significantly higher in healthy adolescents compared to those with TCC	[109]
Significantly strong association of serum DEHP with the rate of DTC in patients with benign thyroid nodules	[111]	Frequency of TCC adolescents in the third group of MCiNP, MCPP, and MiBP lower than that of healthy controls	[109]
Geometric mean of urinary levels of MMP, MEHHP, and MEHP higher in patients with TC and benign nodules than in heathy subjects	[114]	No significant changes in serum DEHP concentration (only exposed subjects) between patients with thyroid nodules and those with DTC	[111]
Highest tertiles of MMP, MEHHP, and MEHP significantly associated with increased risk of TC	[114]	No significant correlation between serum DEHP concentration and TSH levels (only subjects not under levothyroxine treatment)	[111]
MMP, MEOHP, MEHHP, and MEHP levels significantly and positively correlated with risk of benign thyroid nodules in both categorical and continuous analyses	[114]	Geometric mean of urinary levels of MBP and MBzP in patients with TC and benign nodules lower than that in healthy subjects	[114]
Effect modification by sex between MEP and risk of benign nodules and between MEOHP and risk of TC	[114]	The second and the third tertiles of MBP significantly and inversely correlated with TC risk and with the risk of benign thyroid nodules as both categorical and continuous variable	[114]
Higher concentration of urinary molar sum of DEHP metabolites in PTC subjects	[115]	At high iodine levels, urinary concentrations of MBP and MEP not significantly associated with increased risk of PTC	[115]
Both the urinary molar sum of DEHP metabolites and concentration of single DEHP metabolites significantly correlated with increased risk of PTC	[115]	Single measurement of urinary phthalate metabolites or differences in methods of measurements of urinary metabolites	[99,102,109,111,114]
Signals of positive association between urinary MBP concentration and TC risk in the multivariable analysis	[115]	Lack of evaluation of exposure to other EDCs, nutrient/iodine intake, sex hormones, and pre-existing thyroid dysfunction	[99,115]
Urinary levels of DEHP metabolites significantly and strongly correlated with a higher risk of PTC regardless of iodine status	[115]	Single-center study	[99,102,109,114,115]
		Lack of correction for urinary creatinine	[99]
		Lack of assessment of the association between phthalate exposure and thyroid function	[109]

Abbreviations: DEHP: di-(2-ethylhexyl) phthalate; DTC: differentiated thyroid cancer; fT3: free triiodothyronine; fT4: free thyroxine; MBP: monobutyl phthalate; MBzP: monobenzyl phthalate; MCiNP: monocarboxy isononyl phthalate; MCPP: mono-3-carboxylpropyl phthalate; MECPP: mono-[2-ethyl-5-carboxypentayl] phthalate; MEHP: monoethylhexyl phthalate; MEHHP: mono (2-ethyl-5-hydroxyhexyl) phthalate; MEOHP: mono (2-ethyl-5-oxohexyl) phthalate; MEP: monoethyl phthalate; MiBP: mono-isobutyl phthalate; MMP: monomethyl phthalate; PTC: papillary thyroid cancer; SCH: subclinical hypothyroidism; TC: thyroid cancer; TCC: thyroid colloid cyst; TPO: thyroid peroxidase.

### 4.2. Thyrotoxicity of Phthalates: The Underlying Mechanisms

Based on experimental studies, numerous mechanisms have been proposed to attempt to explain the disrupting effects on the thyroid (reviewed in [120]).

**Figure 3:**
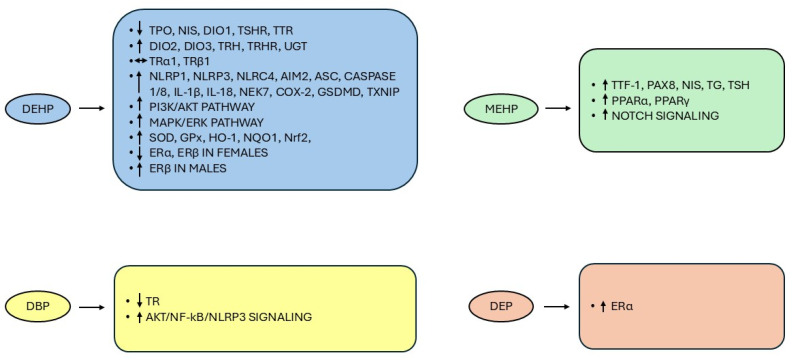
Summary of known effects of phthalates and some of their metabolites on molecules potentially involved in thyroid dysfunction. The up, down, and horizontal arrows indicate upregulation, downregulation, and absence of significant effects, respectively (see text for details). Abbreviations: AKT: protein kinase B; AIM2: absent in melanoma 2; DBP: dibutyl phthalate; DEHP: di-(2-ethylhexyl) phthalate; DEP: diethylphthalate; DIO: deiodinase; ER: estrogen receptor; GPx: glutathione peroxidase; HO-1: heme oxygenase; MEHP: mono(2-ethylhexyl) phthalate; NF-κB: nuclear factor kappa B; NIS: sodium-iodide symporter; NLRC4: NLR family caspase activation and recruitment domain (CARD) domain-containing protein 4 NLRP1/3: NOD-like receptors (NLRs) family pyrin domain containing protein 1/3; Nrf2: nuclear factor-erythroid 2 related factor; NQO1: NAD(P)H quinone dehydrogenase 1; PAX8: paired box gene 8; PPAR: peroxisome proliferator-activated receptor; SOD: superoxide dismutase; TG: thyroglobulin; TPO: thyroid peroxidase; TR: thyroid receptor, TRH: thyrotropin-releasing hormone; TRHR: thyrotropin-releasing hormone; TSH: thyroid stimulating hormone; TSHR: thyroid stimulating hormone; TTF-1: thyroid transcription factor-1; TTR: transthyretin; UGT: uridine 5′-diphospho-glucuronosyltransferase.

1. Exposure to phthalates may interfere with the HPT axis at various biological levels. DEHP can affect TH biosynthesis by reducing serum levels of TPO, NIS, and hepatic DIO1 mRNA level (with consequent decrease in T3 production) and upregulating DIO2 and DIO3 in rats [116]. DEHP also inhibited gene and protein expression of TTR, reducing the biological effects of THs on target tissues [121,122]. Upon DEHP exposure in rats, mRNA expression of TSH receptors (TSHR) was downregulated, with concomitant upregulation of TRH and TRH receptors (TRHR) and no changes in TRα1 and TRβ1 mRNA expression, which collectively reduced the secretion of pituitary TSH and the consequent production of THs [23,121,123]. Conversely, prior studies based on reporter gene assays showed that high concentrations of DEHP, DBP, and DnBP exerted TR antagonistic activities [124,125]. Furthermore, in contrast with [121], zebrafish larvae exposed to MEHP were characterized by induced transcription of genes involved in thyroid development (thyroid transcription factor-1—*TTF-1* and paired box 8—*PAX8*; the main transcription factors involved in the expression of thyroid differentiation marker genes such as *NIS*, *TSHR*, and *TG* [126]) and TH synthesis (*NIS*, *TG*, and *TSH*) [122]. DEHP can further induce the gene expression of uridine 5′-diphospho-glucuronosyltransferases, the liver enzymes that catalyze conjugation of THs with glucuronic acid, in both rats and zebrafish embryos/larvae, thereby promoting catabolism and excretion of THs [121,122].
2. In the thyroid follicular epithelial cell line, DBP exposure can play a central role in thyroid inflammatory damage through the activation of protein kinase B (AKT)/nuclear factor kappa B (NF-κB)/NOD-like receptors (NLRs) family pyrin domain-containing protein 3 (NLRP3) signaling [127]. In particular, the canonical NF-κB pathway, which controls several aspects of cell growth and survival, inflammation, and immune response, is implicated in the regulation of thyroid physiology, participating in the expression of various thyroid-specific genes, including *NIS*, *PAX8*, *TG*, *TPO*, and *TTF-1*, and also contributes to the development of several neoplasms, including TC, enhancing the proliferation and viability of thyroid neoplastic cells and their potential to migrate and colonize distant sites [128,129,130]. The canonical NF-κB signaling can be activated by a wide range of stimuli such as ROS, inflammatory cytokines (e.g., tumor necrosis factor-alpha (TNF-α), interleukin (IL)-6, and IL-1β), growth factors, radiations, infections, and oncogenic stresses and is in turn responsible for the activation of the NLRP3 inflammasome and the transcriptional induction of pro-inflammatory cytokines (pro-IL-1β and pro-IL-18, converted to mature forms by caspase-1, one of the NLRP3 components) and chemokines (TNF-α) [130]. Wu et al. [131] documented that DEHP exposure induced thyroid injury through the inflammasome pathway, with increased mRNA and protein levels in rat thyrocytes of: NLRP1, NLRP3, NLR family caspase activation and recruitment domain domain-containing protein 4 and absent in melanoma 2, the last two inflammasomes functioning as innate immune machines against bacterial and viral infections and in response to cell stress [132,133]; apoptosis-associated speck-like protein containing a CARD, which recruits and activates caspase-1 [134]; caspase-1 and -8 (the latter activated within NLRP3 and having a fundamental role in controlling apoptosis, pyroptosis, and necroptosis in the non-canonical inflammasome pathway [135,136]); IL-1β and IL-18; NIMA-related kinase 7 (a serine/threonine kinase promoting the mitotic cell cycle and involved in NLRP3 assembly and activation upon sensing mitochondrial and cytosolic ROS [137,138]); cyclooxygenase-2, which mediates increase in NLRP3 inflammasome activation [139]; gasdermin D, another component of inflammasomes required for pyroptosis, a type of programmed cell death involving an early destruction of cell membrane, and secretion of IL-1β [136]; thioredoxin-interacting protein (TXNIP), a master regulator of cellular redox that exerts an inhibitory action on thioredoxin, one of the major thiol antioxidants, with consequent increase in ROS concentration, and activates NLRP3 [138,140]. Importantly, N-acetyl cysteine, a scavenger of intracellular ROS, inhibited the increase in protein expression of TXNIP, NLRP3, caspase-1, and IL-1β, the activation of DEHP-induced inflammasome activation and pyroptosis, and the TXNIP-NLRP3 inflammasome pathway [131].
3. The association between exposure to certain phthalates and TC could involve the TSH/TSHR pathway, which plays a pivotal role in the proliferation and differentiation of thyroid cells, being involved in the expression of *TG*, *TPO*, and *NIS* via the upregulation of *TTF-1* and *PAX8* [121,141]. Dong et al. [142] demonstrated that a 6-month treatment with DEHP resulted in increased mRNA and protein expression levels of NIS, TPO, TG, and TSHR with a concurrent downward trend of hypothalamus TRHR mRNA and serum TRH levels and an overall decrease in circulating TH levels. This finding is probably attributable to DEHP-induced imbalance of TSH-TSHR-cAMP-protein kinase. A signaling pathway that promotes an increase in TTF-1 and TSHR expression and activity and subsequently increased expression of NIS and TPO in thyroid tissue and secretion of THs generates a negative feedback regulation leading to inhibition of TRH and TSH production [142,143]. Furthermore, DEHP may increase TSHR expression with the subsequent activation of downstream effectors of the phosphatidylinositol-3 kinase (PI3K)/AKT and mitogen-activated protein kinase/extracellular signal-regulated kinase (MAPK/ERK) pathways, which regulate cell growth, differentiation, and apoptosis and whose genetic alterations have been associated with the onset and progression of TC [144,145,146]. DEHP may directly activate the two pathways in both rat thyroid and human thyroid follicular epithelial cell lines, likely by inducing increased production of ROS, which is accompanied by reduced activity of glutathione peroxidase (GPx) and superoxide dismutase (SOD) and increased levels of malondialdehyde [23].
4. MEHP can induce tumorigenesis through activation of the nuclear transcription factor peroxisome proliferator-activated receptor-alpha (PPARα), a ligand-activated transcription factor belonging to the nuclear receptor superfamily and able to bind the retinoid X receptor to activate the transcription of selected genes [147]. The same phthalate metabolite can also activate PPARγ, which, in addition to regulating adipocyte differentiation and metabolism of lipids and glucose [148,149], when overexpressed, has been associated with more aggressive features in TC, i.e., increased cell growth and tendency to generate metastasis, and appears to promote the transition from differentiated to TC undifferentiated histological types with a consequent worse prognosis [150]. Conversely, depletion of PPARγ inhibited in vitro cell growth and reduced tumor growth in vivo xenograft models [148]. Indeed, PPARγ overexpression suppresses the PTEN-inhibitory action on AKT, resulting in activation of the PI3K signaling pathway, while PAX8-PPARγ rearrangement, a chromosomal translocation between the 5′ portion of *PAX8* and the coding exons of *PPARγ* and detected in up to 60% of cases of follicular thyroid cancer (FTC), may directly induce the MAPK pathway [142,151].
5. In neuroblastoma cells, MEHP also increased the protein expression of the Notch signaling cascade, thereby inducing cell proliferation and inhibiting apoptosis [152]. Notch signaling, initially identified in a mutant strain of *Drosophila melanogaster* and highly conserved throughout evolution, consists of four transmembrane receptor isoforms (Notch1–4) capable of binding to five different ligands (Delta-like-1, -2, -4, Jagged1, Jagged2) and participating in a variety of biological processes like cell fate (proliferation, differentiation, survival) and tissue homeostasis [153,154]. Notch signaling is also considered a key player in carcinogenesis, acting as either an oncogene or a tumor suppressor in various malignancies, including different TC histotypes, thus suggesting that its apparent dual role is cell type and context-dependent [152,153]. Among DTC, while PTC is generally characterized by high levels of Notch signaling, which is involved in promoting cell differentiation, in other less differentiated TC subtypes, namely FTC, medullary thyroid cancer, and anaplastic thyroid cancer, the low levels of Notch receptors are associated with more aggressive properties of neoplasms [153]. As recently demonstrated by Mosteiro and co-authors [155], the thyroid is strictly dependent on Notch for its homeostasis, and Notch inhibition promoted the destruction of thyroid function, with upregulation and downregulation of selected genes, including a decrease in *PAX8* expression in both mouse and human thyroids. Furthermore, Notch suppression affected mitochondrial activity, thereby resulting in decreased levels of ROS and, given their essential role in TH synthesis, hypothyroidism [155]. Of note, DEHP exposure induced testicular toxicity in vivo and in vitro by inducing oxidative stress as revealed by the upregulation of SOD, GPx, heme oxygenase, NAD(P)H quinone dehydrogenase 1 and the transcription factor nuclear factor-erythroid 2 related factor (Nrf2), which overall regulate the redox balance in human cells [156]. Importantly, although the relationship between Notch and Nrf2 is controversial, the authors suggested that the toxic effects exerted by DEHP in the testis were due to a pro-oxidant mechanism that activates the Nrf2-mediated Notch1 pathway [156].
6. Certain phthalates, such as DEHP, DEP, DBP, DiNP, and BBP, appear to act as estrogen agonists in experimental studies [157,158,159,160]. 17β-estradiol (E2) is the most potent physiological estrogen in vertebrates whose effects are not only restricted to reproduction, being implicated in a multitude of actions including cell growth and differentiation even in non-reproductive tissues [158,161]. E2 binds to the nuclear estrogen receptors (ERs) ERα and ERβ, and the formed complexes promote the transcription of genes containing estrogen response element sequences, which also may lead to the development of several types of estrogen-responsive cancers [158,161] including breast [162], endometrial [163], and ovarian cancers [164]. Furthermore, consistent with the 3- to 4-fold prevalence of both benign and malignant thyroid tumors in females compared to males, both ER isoforms have been detected in goiter tissues and TC, and it has been hypothesized that changes in the ERα:ERβ subtype ratio expression may enhance cell proliferation (ERα dominance) or induce cell apoptosis and tumor suppression in PTC (ERβ dominance) [165,166]. Alternatively, the non-genomic signaling of E2, which occurs through its membrane receptors, stimulates the activation of MAPK and PI3K pathways, which represent the major signaling cascades involved in thyroid tumorigenesis [165,167]. DEP treatment may activate ERα without binding the receptor in human breast cancer cells and induce cell proliferation to the same extent as E2 but through AKT activation alone without any effect on the MAPK/ERK signaling cascade [158]. Besides, perinatal exposure to DEHP affected the pituitary ERα and ERβ expression pattern from prepubertal and adult female rats and reduced the number of both the endocrine cells, lactotrophs and somatotrophs, expressing ERα and ERβ, suggesting that DEHP exposure can lead to cell deregulation in the pituitary gland, promoting an imbalance in ER expression [159]. Conversely, perinatal DEHP exposure in male rats induced changes in the pattern of both ER isoforms in prepubertal rats that became relevant only in adulthood, clearly indicating a sex-dependent impact of DEHP on pituitary ERs due to compensatory mechanisms that occur in the prepubertal age but are not sufficient to balance DEHP effects in adults [160].
7. The reported data therefore supports an interaction of certain parent phthalates and phthalate metabolites with the HPT axis, with the consequent possibility of thyroid dysfunction, injury, and even cancer through multiple biological processes (Figure 3), although further studies evaluating the effects of phthalate mixtures at environmental doses would provide more valuable information to translate findings from the experimental models into real-world exposure-outcome relationships in humans.

## 5. The Effects of Non-Phthalate Plasticizers on the Thyroid Gland

Although NPP have been shown to have multiple adverse effects on human health (reviewed in [71]), there are currently an extremely limited number of studies from experimental and human research on the associations between exposure to NPP and thyroid dysfunction. The only epidemiological study evaluating this relationship is a Swedish population-based cohort study involving 1996 pregnant women, which reported a significantly positive association between cyclohexane-1,2-dicarboxylic acid-mono(oxo-isononyl) ester and TT3 levels, a significantly lower TT4/TT3 ratio, and a significantly higher TT3/fT3 ratio, overall suggestive of interference with TH metabolism [98]. However, as observed in Section 3, the cross-sectional design and the single measurement of exposure are two major limitations of this study [98].

A series of experimental studies analyzed the impact of different NPP on the endocrine system of the Japanese rice fish, or medaka (*Oryzias latipes*) model [168,169,170,171]. At the lowest concentration treatment, DEHA significantly enhanced the expression of the thyroid-stimulating hormone beta subunit gene (*tshβ*), while significantly reducing the expression of *dio2* at higher concentrations [171]. In addition, unlike DEHP, DEHA did not significantly affect the expression levels of *tshβ*-like, *trα*, and *trβ* at any concentration tested [171]. The thyroid axis and THs are the major regulators of metabolism, growth, skeleton development, thermo- and osmoregulation, and swim bladder formation in fish, and in zebrafish, *dio2* suppression, resulting in decreased T3 levels, is correlated with defects in swim bladder inflation during larval development [171,172]. Indeed, DEHA caused swim bladder inflation failure and, like DEHP, decreased total body length and eye size at all exposure concentrations, similar to other compounds with endocrine-disrupting properties [171,173,174]. Treatment with the non-phthalate ATBC plasticizer, performed on both zebrafish and Japanese medaka, also affected thyroid-related gene expression, accompanied by a failure of swim bladder inflation in both species [168]. In detail, ATBC suppressed *tshβ*, *trα*, *dio1*, and *dio2* without altering *trβ* expression in the two highest exposure concentration groups of zebrafish [168]. On the other hand, in Japanese medaka, the compound only suppressed the expression of *trα*, *trβ*, and *dio2* in different exposure-concentration groups with no significant changes in the expression of *tshβ* and *dio1* [168]. The same authors showed that also bis(2-ethylhexyl) sebacate (DEHS) exposure was associated with thyroid-disrupting effects in the Japanese rice fish, namely a lower expression level of *tshβ*-like and *trβ* in the lower-concentration group compared to the control group, without any significant change in the expression levels of *tshβ*, *dio1*, and *trα* [170]. In line with [171], DEHS-induced *dio2* suppression led to swim bladder inflation failure but had no effects on swimming performance, although the assessment, performed one day after hatching, should be re-evaluated [170]. Exposure to diisobutyl adipate (DIBA) also disrupted thyroid function in Japanese medaka by markedly increasing *tshβ*-like and *dio1* and reducing *trα* and *trβ* expression compared to the control group and leaving *dio2* unchanged [169]. In addition, all DIBA exposure concentrations were associated with a significant decrease in total body lengths at 1 day after hatching and reduced swimming performance at all concentrations except the lowest [169]. A molecular study aimed at comparing the structural binding of DINCH, ATCB, DEHA, and DEHP against the TRα binding site demonstrated that all ligands formed stable complexes with TRα, and the estimated binding energy was similar (for DEHP) or even higher (for DINCH and ATBC) than that estimated for the native ligand T3 [71]. Furthermore, the binding energy values for DINCH and ATBC were 19% and 17% higher than those of DEHP, respectively [71]. In an earlier study, DEHA also induced thyroid disruption by inhibiting the proliferation of rat pituitary GH3 cells, like other phthalate compounds [175].

Although cautiously due to the extremely low amount of data, NPP appear to affect thyroid function like phthalates and could potentially lead to thyroid damage, which should therefore be further explored in animal models and observational human studies on different population groups.

## 6. The Dietary Source of Phthalates and Non-Phthalates: Current Status and Future Strategies

### 6.1. Edible Compounds and Plasticizers: Current Status

The chemical structure of phthalates and NPP determines many of their physical and chemical properties. Although their vapor pressure is generally low, they can still exist in the vapor phase [176]. In addition, the lipophilic nature of plasticizers influences their ability to migrate and distribute in the environment, with contaminated food likely representing the primary source of plasticizer exposure to humans [176]. A number of edible compounds are largely affected by the presence of plasticizers. Notably, meat and animal-derived products are well known for being a source of phthalates in the dietary framework, with a specific relationship with the fat content in the meat sample and the period of storage [177]. On the other hand, according to a Taiwanese study, unpackaged meat would not present significantly harmful levels of phthalates, which confirms the main risks to be related to the packaging rather than to the production process or feeding material for the animals [178,179]. Besides, especially when such products are consumed within fast foods, the discharge of traditional and novel plasticizers can also occur via the gloves used to prepare and handle such meals [78,180].

Also of animal origin are dairy products, including milk, which, being classified as high-fat food, is greatly affected by phthalate contamination, along with the whole production chain, and in particular in the processing of the milk into the final product [181], including creams (mostly affected) and light milk (affected the least), and in the packaging process [181], with DEHP normally considered the main plasticizer found in the samples [181,182], whereas the feeding process of the cattle showed an increase in phthalates just in a few cases [183]. Vegetables represent an important source of food throughout the globe, with some cultures relying on them for the majority of their daily subsistence. In such compounds, the concentration of phthalates has a different source, being mainly represented by compounds concentrated in the soil, mainly derived from film mulching, wastewater irrigation, fertilizers, and sewage sludge [184], which are, more recently, increasingly rich in novel plasticizers [185]. However, as also observed for animal-based compounds, even among vegetables phthalates are mainly present in parts of the plant richer in lipids, including roots, probably due to their tendency to accumulate hydrophobic compounds [186]. In addition, a further accumulation of phthalates may occur from the employment of plastic films and items during the production. Interestingly, when dealing with greenhouse-grown vegetables, which in turn are richer in DBP and DEHP than those grown outdoors, it seems that the highest degree of phthalate accumulation occurs in the leaves rather than in soils, with a certain correlation between DEHP concentration and cultivation time in greenhouses, possibly due to the use of plastic films, whereas DBP was associated with the use of fertilizers and pesticides [187]. The same problem is also seen in soups and other liquid foods. In particular, high-temperature soups are often packaged in plastic-made wraps, thus being associated with an increased exposure to phthalates and also bisphenol A (BPA) and other compounds [188,189]. Of utmost importance in the diet are oils and dressings. Depending on the region of living and the culture of consumers, different condiments are being used. For example, olive oils, prevalent in the Mediterranean diet, are produced from olives that present higher concentrations of DiBP, DBP, and DEHP when harvested in mills than when harvested in the orchard, possibly suggesting the source of contamination during harvesting and transport to the mill, beyond the contact with other production means, including pipes and other materials [190]. Another research study found higher concentrations of phthalates in olive oils than in other oils, with press-extracted oils more contaminated than the others and a high contamination level for oils derived from perennial plants, in turn with a greater potential for bioaccumulation [191], whereas chemical and physical refining of seed oils could lead to a decreased concentration of phthalates [192]. In commercialized oils, it has been postulated that the high levels of phthalates could be due to the use of adhesives, lacquers, and printing inks in PET bottle packaging [193]. Notably, eating ultra-processed foods and meals prepared in fast-food restaurants contributes to human exposure to both phthalates and NPP, such as DEHT, suggesting the need to plan individual and regulatory strategies to promote consumption of home-prepared fresh foods [78,194,195,196,197,198].

When it comes to beverages, mineral water can be contaminated mainly due to the presence of phthalates in the bottle. In particular, the raw material quality and the fabrication technology for bottles and caps, the water sources contamination with decomposed plastic, and the cross-contamination of bottling factories represent the major threats in this regard [78,186,187,188,189,190,191,192,193,194,195,196,197,198,199,200,201]. However, other factors may influence the presence and concentration of phthalates in mineral water, including pH, storage time, and temperature, as well as exposure to sunlight [199,200,202,203]. Compared to mineral water, soft drinks are more (5–40 times) susceptible to contamination due to their strong acidity and carbon dioxide content, which favors the migration of phthalates [204]. Also, additives commonly employed to enhance the qualities and appearance of sports drinks can represent another relevant source of contamination [205]. Finally, coffee and tea can be affected by contaminants contained in capsules (mainly biodegradable ones) [206] and by packaging plastics in general [207]. Finally, alcoholic beverages are not free from contamination since their ethanol content makes them susceptible to the presence of phthalates in their samples, acting as a solvent for their extraction [208]. In this way, plasticizers can remain in contact with the beverage during the entire production chain, as it happens with wine, from the transportation of fruit to the pressing to the storage, all processes that involve the use of equipment that discharge phthalates, including pumps, hoses, tools for filtration, and final packaging [209]. Besides, compared with wines, a higher percentage of spirit samples exceeded the specific migration limits for FCMs, with the epoxy resin coatings of wine storage and fermentation vats as the main contaminants [210], while the higher ethanol concentration can partially explain this phenomenon [211,212].

### 6.2. Future Strategies to Reduce Plasticizers in Food

It is well known that the massive use of plasticizers in the food industry is strongly related to the final goal of maintaining the sensory properties of a given compound, making it edible and appealing to the consumer for a long time. However, such characteristics should be ensured in a balanced manner with the proper use of compounds that are not harmful to the health, safety, and well-being of the consumers themselves. In this, two directions should be tailed. First, the development of novel packaging tools made up of non-plastic raw materials, therefore safe and secure in terms of phthalate discharge. Many groups have already adopted paper-based or plant-based alternatives, also relying on production wastes of agricultural production waste, putting into practice the basic concepts of the circular economy, with mixed success [213,214,215]. On the other hand, a joint effort should be performed to optimize the shelf life of products using non-destructive methods to assess their organoleptic properties. This effort should be paid for by professionals and companies using electronic devices (e-nose, e-tongue, e-eye), whose retrievals, joined with the feedback from trained panelists, can determine the possibility of extending the shelf life of a product even beyond its natural “best before” date without risk to human health, while reducing food waste [216].

## 7. Main Issues Related to the Migration Process from FCMs to Foodstuffs

The chemical migration from packaging into food and the environment is a diffusion-driven process influenced by multiple variables [27]. This mass transfer is governed both by kinetic and thermodynamic factors and can be expressed using Fick’s diffusion model [217]. Key parameters affecting migration include the initial concentration of migrants, diffusion coefficients, and partition coefficients between packaging and food or the environment [218]. Migration behavior falls into three categories based on diffusion coefficients: minimal migration, time-independent migration, and food-contact-dependent migration [219].

**Figure 4:**
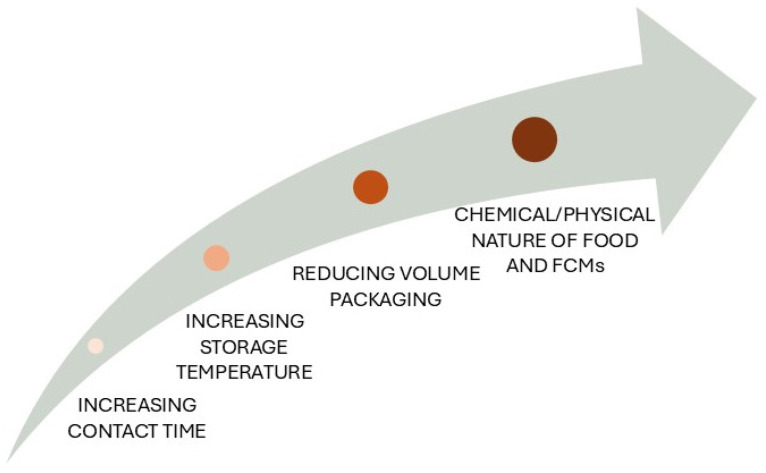
Schematic representation of checkpoints within the hypothalamic-pituitary-thyroid axis on which plasticizers may exert their effects (see text for details). Abbreviations: FCMs: food contact materials.

Factors influencing FCM-dependent migration (Figure 4) include molecular properties of both FCMs and foodstuffs, FCMs/foods interactions, and resistance due to barrier layers [218]. Additionally, migration rates increase with extended contact time, elevated temperatures, larger contact areas, and the chemical aggressiveness of food [220].

Higher migrant concentrations in packaging and variations in polymerization degree also impact migration [218]. Understanding these factors is essential for designing safer packaging materials that minimize chemical transfer into food products.

Finally, some main recycling issues should be mentioned, as the use of recycled plastics in food-contact applications presents potential risks due to the migration of contaminants into packaged food. These contaminants may originate from various sources, including the improper use of plastic materials, non-food contact applications, and substances such as PVC, polyamides, polyolefins, adhesives, and label residues [221,222]. Chemicals employed in the recycling process and degradation products of plastic materials themselves can also contribute to contamination [223]. Furthermore, food components from the initial use, such as oils, fats, and flavor compounds, may be absorbed into the plastic and subsequently released during recycling [224].

In this context, as reported in [176], it could be useful to identify some good practices to adopt both at a regulatory level and in households to reduce as much as possible the migration of plasticizers from FCMs to foods (Table 3):

## 8. Conclusions

**Figure 5:**
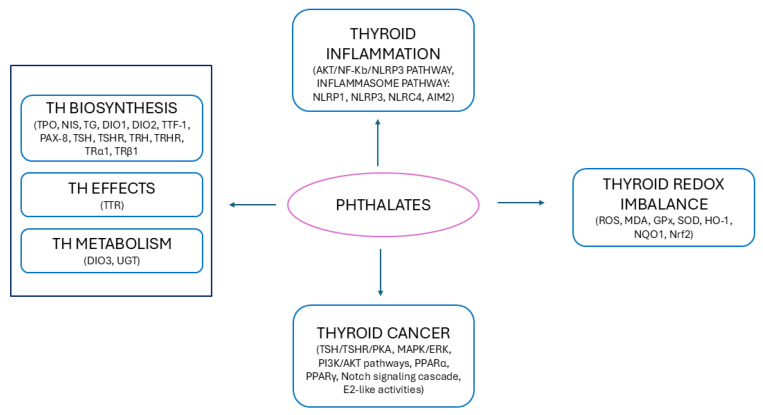
Schematic summary of multiple interactions of phthalate with the hypothalamic-pituitary-thyroid axis and the molecules or pathways involved in these processes (see text for details). Abbreviations: AKT: protein kinase B; AIM2: absent in melanoma 2; DIO: deiodinase; E2: 17β-estradiol; GPx: glutathione peroxidase; HO-1: heme oxygenase; MDA: malondialdehyde; NF-κB: nuclear factor kappa B; NIS: sodium-iodide symporter; NLRC4: NLR family caspase activation and recruitment domain (CARD) domain-containing protein 4 NLRP1/3: NOD-like receptors (NLRs) family pyrin domain containing protein 1/3; Nrf2: nuclear factor-erythroid 2 related factor; NQO1: NAD(P)H quinone dehydrogenase 1; PAX8: paired box gene 8; PKA: protein kinase 1; PPAR: peroxisome proliferator-activated receptor; ROS: reactive oxygen species; SOD: superoxide dismutase; TG: thyroglobulin; TH: thyroid hormone; TPO: thyroid peroxidase; TR: thyroid receptor; TRH: thyrotropin-releasing hormone; TRHR: thyrotropin-releasing hormone; TSH: thyroid stimulating hormone; TSHR: thyroid stimulating hormone; TTF-1: thyroid transcription factor-1; TTR: transthyretin; UGT: uridine 5′-diphospho-glucuronosyltransferase.

Limited evidence supports the thyroid disrupting properties of phthalates through effects on thyroid function at various biological levels (Figure 5).

In particular, these compounds, which may interact with the human body through multiple sources (e.g., food containers, personal care products, medical devices) and routes of exposure (ingestion, dermal contact, inhalation, and placenta and breastfeeding for fetuses and infants, respectively), exert toxicity in various organ systems and also appear to interfere with thyroid function. Indeed, certain phthalates and/or their metabolites may affect TH levels in a sex- and age-specific manner, increasing or decreasing the concentration of circulating bound hormones with actions involving a number of genes encoding master transcription factors and enzymes that catalyze the synthesis, transport, and metabolism of THs. Given the relevant role of THs in children’s neurodevelopment, biomonitoring of pregnant women with high exposure to these compounds would be recommended. In this context, future large prospective studies performing multiple measurements of phthalate metabolites and thyroid parameters at different time points of pregnancy would provide more reliable estimates. The assessment of the effect of co-exposures to phthalate mixtures, in addition to the role of iodine intake and thyroid autoantibodies status, could also improve the understanding of associations between phthalate exposure and thyroid dysfunction. Notably, in addition to phthalates, people are constantly exposed to plastics due to the large expansion of plastic manufacturing and, consequently, to a variety of associated harmful chemicals including bisphenols, polybrominated diphenyl ethers, polybrominated biphenyls, and heavy metals, known EDCs with thyroid-disrupting properties, as well as to micro- and nanoplastics, which result from plastic degradation containing EDCs, which absorb EDCs and may enhance their bioaccumulation in humans [225,226]. Therefore, the toxic effects on the thyroid observed for phthalates in human studies cannot rule out the possible contribution of micro- and nanoplastics and/or EDCs, and future studies should also assess the effect of multiple exposures to different contaminants. Furthermore, phthalates appear to be also associated with an increased risk of thyroid injury and the development of benign and malignant thyroid nodules, corroborated by the results from experimental studies, although the few epidemiological studies performed so far, the small number of participants, and the cross-sectional design do not allow definitive conclusions to be drawn, indicating the need for further large, multicenter studies. As for NPP, despite the paucity of experimental data and the near lack of human research, accumulating evidence suggests a potential role of these chemicals in increasing the risk of thyroid dysfunction, raising questions about their greater safety compared to phthalates. Therefore, given the widespread use of NPP, in vivo and human studies are urgently needed, and their possible thyroid-disrupting effects should also represent a priority for international authorities and environmental agencies, also considering that children and pregnant women living in urban areas are particularly at risk of exposure.

On the other hand, an analysis of the available literature on this topic [27,217] reveals key gaps in current experimental designs for assessing chemical migration from FCMs to foodstuffs, which do not allow for obtaining consistent and conclusive evidence. One major issue is the unclear chemical composition of FCMs, particularly in multi-layered packaging, making it difficult to identify potential migrants, mainly for biodegradable and recycled materials. Additionally, baseline contamination of water, food, and beverages before packaging is often overlooked, complicating the assessment of migration levels. While food simulants are used to model migration, their accuracy remains questionable, and predictive mathematical models require further validation. Other critical factors include the influence of FCM size, storage temperature, and contact time on migration, which are often inconsistently reported. There is a pressing need for internationally standardized testing methods and improved risk management strategies. Besides, long-used substances should be reassessed for endocrine-disrupting properties using modern toxicology principles. Health risk assessments must also consider non-intentionally added substances, such as plastic oligomers, which may act as endocrine disruptors [225]. Furthermore, the cumulative effects of chemicals below their individual “no observed adverse effect levels” should not be ignored, as even low-dose exposures can have adverse health effects.

Overall, the primary source of plasticizers in food comes from the raw materials used in packaging, which, if not properly selected or misused in household practices, can pose significant risks to consumers’ health and well-being. In that, the development of new packaging materials, also fostering a circular economy, and the correct determination of the shelf life of edible compounds can be fundamental for the success of products on the table, increasing the appeal for the consumer, and reducing the use of both traditional and novel plasticizers for their preservation, to the ultimate benefit of the consumers themselves and their health.

## Figures and Tables

**Table 1:** Clues and pitfalls in the relationship between phthalate exposure and thyroid dysfunction.

Clues	Reference	Pitfalls	Reference
Urinary MEHP and MEHPP levels inversely correlated with serum levels of TT4	[91]	Differences across adults, pregnant women, and children in the associations between phthalate metabolites and levels and thyroid parameters	[91]
Urinary MEOHP concentration positively associated with serum levels of TSH	[91]	Single measurement of urinary phthalate metabolites	[3,91,94,98,99,100]
Each 10-fold increase in maternal urinary MEP negatively associated with serum TT4	[92]	Phthalate metabolites and THs measured by different methods in the studies included	[91]
Each 10-fold increase in average maternal urinary MBzP inversely correlated with cord serum TSH	[92]	Lack of correction for urinary creatinine and/or specific gravity	[91,93,94,97,99]
Phthalate index (MEP and MCPP the most contributors) associated with decreased maternal TT4	[92]	Possibility that the analytical method cannot detect the non-linear relationship between phthalate exposure and thyroid function	[91,94]
Phthalate index (MBzP and MiBP the most contributors) associated with decreased cord serum TT4 and TSH	[92]	Conflicting results on the modifying effect of iodine in the relationship between phthalate exposure and thyroid parameters	[92,93,94]
IQR increase in several phthalate metabolites significantly associated with increased levels of TT3 in each study visit	[93]	The use of WQS does not consider the direction of association of single metabolites and assumes both a linear relationship between exposure and outcomes and the absence of interactions between metabolites within cumulative index	[92]
IQR increase in MCOP and MCPP significantly associated with increased levels of fT4 and TT4	[93]	Lack of evaluation of BMI, iodine intake, thyroid autoantibodies, or pre-existing thyroid dysfunction	[3,92,93,94,97,98,99,101]
Measurements of phthalates and THs at two times/multiple points during pregnancy	[92,100]	Differences in the timing of sample collection during pregnancy and consequent inaccuracy in TH measurements due to variations in TH levels at various gestational age	[92,93,94,98,99]
Factor 1 (MiBP, MnBP, and MBzP as the most contributors) significantly associated with increased levels of TT3 and fT3 in pregnant women	[94]	Possibility of residual bias by the oversampling of mothers with ADHD children	[94]
Significantly inverse association between factor 1 (MiBP, MnBP, and MBzP the most contributors) and ft3 and TT3 in pregnant women with sufficient iodine intake	[94]	Cross-sectional design	[3,91,92,94,97,99]
Factor 2 (molar sum of DEHP and DiNP metabolites the most contributors) significantly associated with decreased levels of fT3 and TT3 independently from iodine intake of pregnant women	[94]	Lack of significant associations between phthalate metabolites and TSH or thyroid autoantibodies	[93,97,98]
Use of factor analysis and of information on the habitual iodine intake to investigate phthalate-thyroid function relationship	[94]	Small number of subjects with positive thyroid autoantibodies	[97]
All phthalate metabolites significantly associated with higher levels of TT3 and, all except for MnBP and MCPP, negatively associated with fT3	[97]	Lack of measurements of albumin, transthyretin, or thyroxine-binding globulin	[98]
All metabolites significantly associated with increased DIO activity	[97]	Small sample size	[99,100,101]
DEHP metabolites significantly and positively associated with TBG levels	[97]	Possibility of recall bias	[100]
Thyroid autoimmunity status able to modify the association between phthalate metabolites and fT3, fT4, TSG, and TBG	[97]	Loss of participants during the follow-up	[100]
Factor (molar sum of MEHHP, MEHOP, MECPP, MnBP, and MBzP) and factor 2 (molar sum of MCOP, MCNP, and MCPP) significantly negatively associated with fT3 and significantly positively associated with TT3, TBG, and DIO activity	[97]		
Higher levels of the molar of DEHP metabolites significantly associated with lower fT4 levels and a higher TSH7fT4 ratio in pregnant women	[98]		
Higher levels of the molar of DiNP metabolites significantly associated with lower TT4 levels and lower TT4/fT4 and TT4/TT3 ratios in pregnant women	[98]		
BBzP and DBP significantly and positively associated with lower TT4/TT3 and fT4/fT3 ratios and with higher fT4/TT4 and fT3/TT3 ratios in pregnant women	[98]		
MEP, MECPP, MEOHP, and molar sum of DEHP significantly associated with a higher risk of SCH during pregnancy	[99]		
MCOP significantly positively correlated with TT3 and TSH in adolescents	[3]		
MCNP significantly inversely correlated with TSH in adolescents	[3]		
Significant negative correlation between levels of MMP and TT3, TT4 and fT4 in children	[100]		
The molar sum of DEHP metabolites, MEHHP and MEHOP, significantly positively correlated with fT4	[100]		
The highest quartile of MEP, MECPP, MEHHP, molar sum of all metabolites, DEHP, two DEP metabolites low- and high-molecular-weight metabolites significantly associated with increased levels of fT4 compared to the lowest quartile	[101]		
The highest quartile of all metabolites (except for MEHHP) and their sum positively associated with increased levels of TSH compared to the lowest quartiles	[101]		
The highest quartile of MEP and molar sum of DBP metabolites positively and inversely correlated with TT3, respectively	[101]		
The highest quartiles (third or fourth) of MEP, MiBP, MnBP, the molar sum of all metabolites, DBP, and low-molecular-weight metabolites significantly associated with a higher TSH/fT4 ratio	[101]		

Abbreviations: ADHD: attention deficit hyperactivity disorder; BBzP: butyl-benzyl phthalate; BMI: body mass index; DBP: dibutyl phthalate; DEHP: di-(2-ethylhexyl) phthalate; DEP: diethyl phthalate; DiNP: diisononyl phthalate; fT3: free triiodothyronine; fT4: free thyroxine; IQR: interquartile range; MBzP: monobenzyl phthalate; MCNP: monocarboxy-isononyl phthalate; MCOP: monocarboxyoctyl phthalate; MCPP: mono-3-carboxylpropyl phthalate; MECPP: mono-[2-ethyl-5-carboxypentayl] phthalate; MEHP: monoethylhexyl phthalate; MEHHP: mono (2-ethyl-5-hydroxyhexyl) phthalate; MEOHP: mono (2-ethyl-5-oxohexyl) phthalate; MEP: monoethyl phthalate; MiBP: mono-isobutyl phthalate; MMP: monomethyl phthalate; MnBP: mono-n-butyl phthalate; SCH: subclinical hypothyroidism; TBG: thyroglobulin; THs: thyroid hormones; TSH: thyroid-stimulating hormone; TT3: total triiodothyronine; TT4: total thyroxine; WQS: Weight Quantile Sum.

**Table 3:** Best practices and recommendations for regulatory bodies and consumers to reduce plasticizers leaching to foods and environment.

Regulatory Bodies Policy	Household Best Practices
Enforce strict limits on plasticizer usage and ensure compliance by manufacturers, with continuous monitoring and periodic updates of regulatory guidelines based on new scientific data.	Avoid extensive reuse of packaging as specific storage conditions that can affect migration, e.g., the aging process of the material as well as mechanical damage due to the repeated use and frequent washing cycles.
Targeted efforts to reduce plasticizer use in vulnerable sectors such as medical devices, children’s playthings, food packaging, and kitchenware, promoting safer alternatives and educating consumers on their correct usage.	Adhere strictly to the usage recommendations provided by the manufacturer on the package label, including guidelines on temperature ranges, cooking and washing utensils, and types of foods to store.
Large-scale investment in biodegradable microorganisms capable of degrading phthalates should be prioritized, alongside research to optimize microbial efficiency.	Reduce exposure to non-dietary sources of plasticizers: thermal paper (dermal route), pharmaceuticals (dermal route/ingestion), cosmetics (dermal route), toys (oral route/ingestion), pacifiers (oral route), surface water (while swimming) (oral route/dermal route), dental fillings (oral route), and dust (inhalation).
Advancements in detection techniques, such as LC and HPLC-MS/MS, are critical to accurately monitor phthalate levels in various environments, enabling better regulatory enforcement and public health protection.	Minimize or eliminate the use of plastic materials in activities for children and toddlers.
Comprehensive strategies involving stricter regulations, citizens’ involvement, public awareness campaigns, and collaboration between actors (governments, NGOs, private sector) are essential to reduce the harmful impacts of phthalates on human health, animals, and ecosystems.	Strictly follow the recycling recommendations provided by the manufacturer on the packaging label and/or by regulatory bodies.
Improve policies to regulate the collection and recycling of food waste, aiming to produce plastic recycled materials for use in FCMs.	

Abbreviations. FCM: food contact materials; HPLC-MS/MS: high-performance liquid chromatography-tandem mass spectrometry; LC: liquid chromatography; NGO: non-governmental organizations.

## Data Availability

No new data were created.

## Abbreviations

AKT	Protein kinase B
ATBC	Tributyl acetyl citrate
BBP	Benzyl butyl phthalate
BBzP	Benzyl butyl phthalate
BMI	Body mass index
CARD	Caspase activation and recruitment domain
DBP	Dibutyl phthalate
DEHA	Di(2-ethylhexyl) adipate
DEHP	Di-(2-ethylhexyl) phthalate
DEHS	Bis(2-ethylhexyl) sebacate
DEHTP	Di(2-ethylhexyl) terephthalate
DEP	Diethyl phthalate
DIBA	Diisobutyl adipate
DiBP	Di-isobutyl phthalate
DiDP	Di-isodecyl phthalate
DINCH	Diisononyl 1,2-cyclohexanedicarboxylic acid
DiNP	Di-isononyl phthalate
DMP	Dimethyl phthalate
DnBP	Di-isononyl phthalate
DTC	Differentiated thyroid cancer
EDC	Endocrine disrupting chemical
ERK	Extracellular signal-regulated kinase
DIO	Deiodinase
E2	Estradiol
ER	Estrogen receptor
fT3	Free triiodothyronine
fT4	Free thyroxine
FCMs	Food contact materials
FTC	Follicular thyroid cancer
GPx	Glutathione peroxidase
HPT	Hypothalamic-pituitary-thyroid
HT	Hashimoto’s thyroiditis
IL	Interleukin
MAPK	Mitogen-activated protein kinase
MBP	Monobutyl phthalate
MBzP	Monobenzyl phthalate
MCNP	Monocarboxy-isononyl phthalate
MCOP	Monocarboxyoctyl phthalate
MCPP	Mono-3-carboxylpropyl phthalate
MECPP	Mono-(2-ethylpentyl-5-carboxy) phthalate
MEHHP	Mono (2-ethyl-5-hydroxyhexyl) phthalate
MEHP	Monoethylhexyl phthalate
MEOHP	Mono (2-ethyl-5-oxohexyl) phthalate
MEP	Monoethyl phthalate
MiBP	Mono-isobutyl phthalate
MMP	Monomethyl phthalate
MnBP	Mono-n-butyl phthalate
NF-κB	Nuclear factor kappa B
NIS	Sodium-iodine symporter
NLRP	NOD-like receptors family pyrin domain containing protein
NPP	Non-phthalate plasticizers
Nrf2	Nuclear factor-erythroid 2 related factor
PAX-8	Paired box 8
PET	Polyethylene terephthalate
PI3K	Phosphatidylinositol-3 kinase
PPAR	Peroxisome proliferator-activated receptor
PTC	Papillary thyroid cancer
ROS	Reactive oxygen species
SCH	Subclinical hypothyroidism
SOD	Superoxide dismutase
T3	Triiodothyronine
T4	Thyroxine
TBG	Thyroxin-binding globulin
TC	Thyroid cancer
TCC	thyroid colloid cyst
TDI	Tolerable daily intake
TG	Thyroglobulin
TH	Thyroid hormone
TNF-α	Tumor necrosis factor-alpha
TPO	Thyroid peroxidase
TR	Thyroid hormone receptor
TRH	Thyrotropin-releasing hormone
TRHR	Thyrotropin-releasing hormone receptor
TSH	Thyroid-stimulating hormone
TSH-β	Thyroid-stimulating hormone beta subunit
TSHR	Thyroid-stimulating hormone receptor
TT3	Total triiodothyronine
TT4	Total thyroxine
TTF-1	Thyroid transcription factor-1
TTR	Transthyretin
TXNIP	Thioredoxin-interacting protein
